# Lower Virus Infections in *Varroa destructor*-Infested and Uninfested Brood and Adult Honey Bees (*Apis mellifera*) of a Low Mite Population Growth Colony Compared to a High Mite Population Growth Colony

**DOI:** 10.1371/journal.pone.0118885

**Published:** 2015-02-27

**Authors:** Berna Emsen, Mollah Md. Hamiduzzaman, Paul H. Goodwin, Ernesto Guzman-Novoa

**Affiliations:** 1 Department of Animal Science, Ataturk University, Erzurum, 25240, Turkey; 2 School of Environmental Sciences, University of Guelph, Guelph, Ontario, N1G 2W1, Canada; Universidade de São paulo, BRAZIL

## Abstract

A comparison was made of the prevalence and relative quantification of deformed wing virus (DWV), Israeli acute paralysis virus (IAPV), black queen cell virus (BQCV), Kashmir bee virus (KBV), acute bee paralysis virus (ABPV) and sac brood virus (SBV) in brood and adult honey bees (*Apis mellifera*) from colonies selected for high (HMP) and low (LMP) *Varroa destructor* mite population growth. Two viruses, ABPV and SBV, were never detected. For adults without mite infestation, DWV, IAPV, BQCV and KBV were detected in the HMP colony; however, only BQCV was detected in the LMP colony but at similar levels as in the HMP colony. With mite infestation, the four viruses were detected in adults of the HMP colony but all at higher amounts than in the LMP colony. For brood without mite infestation, DWV and IAPV were detected in the HMP colony, but no viruses were detected in the LMP colony. With mite infestation of brood, the four viruses were detected in the HMP colony, but only DWV and IAPV were detected and at lower amounts in the LMP colony. An epidemiological explanation for these results is that pre-experiment differences in virus presence and levels existed between the HMP and LMP colonies. It is also possible that low *V*. *destructor* population growth in the LMP colony resulted in the bees being less exposed to the mite and thus less likely to have virus infections. LMP and HMP bees may have also differed in susceptibility to virus infection.

## Introduction

The parasitic mite *Varroa destructor* has become the most serious health problem of the western honey bee, *Apis mellifera*, worldwide. This mite is one of the factors associated with the unprecedented loss of honey bee colonies recently experienced in parts of Europe and North America [1–4]. Recent studies suggest that *V*. *destructor* may be so harmful to honey bees not only because of its feeding on the bee's haemolymph, but also because it transmits and favors the multiplication of honey bee viruses. Increases in the incidence and levels of several honey bee viruses have been observed with *V*. *destructor* as an inducer/or vector of several viruses [5].

The viruses most commonly found in surveys of honey bee colonies worldwide, are deformed wing virus (DWV), acute bee paralysis virus (ABPV), sac brood virus (SBV), black queen cell virus (BQCV), Kashmir bee virus (KBV) and Israeli acute paralysis virus (IAPV) [5–8]. DWV, ABPV and KBV have been associated with cases of bee mortality [9–12], and IAPV has been related to the so-called Colony Collapse Disorder [13]. DWV in particular, has been linked with winter bee mortality in recent studies [14].

It is well known that honey bee genotypes vary in their resistance to *V*. *destructor* [15–17]. However, not much research has been done to show whether honey bee genotypes differ in susceptibility to viruses or if differences in susceptibility are affected by the developmental stage of the bee [18, 19].

The objective of this study was to determine and compare the prevalence and relative amounts of DWV, IAPV, BQCV, ABPV, SBV and KBV in brood and adult honey bees between colonies with either low or high *V*. *destructor* population growth. These colonies had previously bee shown to differ in grooming behavior and *V*. *destructor* reproduction rates [20, 21].

## Materials and Methods

### Assessment of *V*. *destructor* population growth

Experiments were conducted at the Honey Bee Research Centre (HBRC) of the University of Guelph, in Guelph, Ontario, Canada. Full description of the methods used to screen for mite population growth in colonies is described elsewhere [20]. Briefly, 152 honey bee colonies headed by Buckfast queens were evaluated twice for the mean daily mite fall counts (i.e., number of mites dropped on sticky boards in a colony per day). The first evaluations were conducted during the last two weeks of April (spring), whereas the second evaluations were done 16 weeks later during the third and fourth week of August (summer). Mite population increases for the 16 weeks between the spring and summer evaluations were obtained and the ten colonies with the greatest difference were designated high mite population (HMP) colonies, and the ten colonies with the smallest difference were designated low mite population (LMP) colonies. Subsequent testing for infestation of adult bees and brood was conducted for these two groups of colonies.

### Source colonies for brood and adult worker bees

One colony each from the HMP and LMP groups was selected as source colonies for brood and adult worker bees. The mite infestation rates of these colonies were 96, 10 and 17 for daily mite drop, number of mites/100 bees and number of mites/100 cells, respectively for the HMP colony, whereas for the LMP colony, these rates corresponded to 6, 1 and 0.7, respectively (20). Frames containing newly capped cells were used for brood experiments, whereas newly emerged bees were utilized for adult bee experiments. To obtain adult bees, frames containing emerging brood were collected from honey bee source colonies and incubated overnight inside screened cages (5 x 28 x 25.5 cm) at 33–35°C, and 60% RH.

### Collection of *V*. *destructor* mites

Adults *V*. *destructor* were obtained from a heavily infested honey bee colony that had not been treated with miticides for at least six months. Mites were harvested by opening capped brood cells and by collecting them using a fine paintbrush. The harvested mites were either used for virus analysis or infestation. For virus analysis, three samples of approx. 100 *V*. *destructor* mites were placed in 1.5 mL microfuge tubes and kept frozen at -70°C until analyzed. For infestation, mites were kept in a Petri dish lined with moist filter paper and allowed to feed upon two white-eyed bee pupae from a non-infested colony. The mites were used within 2 h after collection.

### Artificial infestation of brood with *V*. *destructor*

To infest brood with *V*. *destructor*, groups of 15 newly-capped brood cells from combs of either a LMP or HMP colony were opened by cutting a thin slit approximately 2 mm long using a sterile blade, and then a single mite was transferred into each cell using a fine paintbrush [22]. The slit was resealed by lightly brushing it with liquid beeswax. For the control, cells were opened with a blade and then resealed without introducing mites in them. The brood frames were incubated at 33–35°C and 60% RH and the test cells were opened and the brood retrieved at 7 days post infestation (dpi). The retrieved brood was stored individually at -70°C in 1.5 mL microfuge tubes until analyzed. This experiment was replicated three times.

### Artificial infestation of adult bees with *V*. *destructor*

To infest adult bees with *V*. *destructor*, ten newly emerged bees were introduced into a Benton queen cage and three cages were used for a total of 30 bees. Two mites were placed on the body of each bee through the screen of the cages using a fine brush. In these cages, the bees were fed queen candy *ad libitum* and given water by spreading drops of water on the screen of the cages twice a day. Bees were incubated at 33–35°C and 60% RH. For the control, newly emerged bees were placed in Benton queen cages and incubated as above without the addition of mites. Surviving workers were collected at 7 dpi and stored individually at -70°C in 1.5 mL microfuge tubes until analysis. This experiment was replicated three times.

### RNA extraction and cDNA synthesis

Total RNA was extracted after homogenizing five bees (brood or adults) or 100 mites as per Chen et al. [23]. The concentration of RNA was determined with a spectrophotometer. For cDNA synthesis, 2 μg of total RNA was reverse-transcribed using the RevertAid H Minus First Strand cDNA Synthesis Kit (Fermentas Life Sciences, Burlington, ON, Canada) and Oligo (dT)_18_ as primer, following the instructions of the manufacturer.

### PCR reactions

Multiplex simultaneous reactions were done combining one set of virus-specific primers with the RpS5 primers. All PCR reactions were done with a Mastercycler (Eppendorf, Mississauga, ON, Canada). Each 15 μl of reaction contained 1.5 μl of 10x PCR buffer (New England BioLabs, Pickering, ON, Canada), 0.5 μl 10 mM of dNTPs (Bio Basic Inc., Markham, ON, Canada), 1 μl each of 10 μM forward and reverse primers for RpS5 and 10 μM forward and reverse primers for ABPV, KBV, DWV, BQCV, SBV or IAPV, 0.2 μl 5U/μl of *Taq* polymerase (New England BioLabs), 1 μl of the cDNA sample, and 7.8 μl of dd H_2_O. For IAPV, ABPV, SBV and KBV, the PCR conditions were 94°C for 3 min, followed by 35 cycles of 30 s at 94°C, 60 s at 55°C and 60 s at 72°C, and a final extension step at 72°C for 10 min. Amplification conditions for DWV and BQCV were the same, except that the annealing temperature was 58°C.

The amounts of ABPV, KBV, DWV, BQCV, SBV and IAPV relative to a bee constitutive control gene were determined by a multiplex reverse transcription-PCR (RT-PCR). Primers for the constitutive honey bee gene were for the ribosomal protein RpS5 gene described by Thompson et al. [24]. Primers for IAPV, ABPV, SBV and KBV were those reported by several authors in previous research [7, 25–27]. The forward primer of Chen et al. [28] for DWV was paired with the reverse primer of Guzman-Novoa et al. [29]. The primers for BQCV were those reported by Benjeddou et al. [30] with slight modifications to obtain a shorter PCR product to avoid possible hairpin loops. The forward and reverse primers were 5′GTCAGCTCCCACTACCTTAAAC and 5′CAACAAGAAGAAACGTAAACCAC, respectively. All primers were obtained from Laboratory Services of the University of Guelph (Guelph, Ontario).

### Separation and quantification of PCR products

PCR products were separated on 1% TAE agarose gels and stained with ethidium bromide. A 100 bp DNA ladder (Bio Basic Inc.) was included in each gel. Images of the gels were captured using a digital camera with a Benchtop UV Transilluminator (BioDoc-It^M^ Imaging System, Upland, CA, USA). To confirm the identity of each PCR product, randomly selected PCR products were purified using the EZ-10 Spin Column DNA Gel Extraction Kit (Bio Basic Canada Inc.) and sequenced at the Laboratory Services of the University of Guelph. The sequences were used to search the GenBank nr database of NCBI by BLASTn, and all of them showed ≥ 96% identity to their respective target virus sequence.

The intensity of the amplified bands was quantified in pixels using the Scion Image (Scion Corporation, Frederick, MD, USA) as per Dean et al. [31]. Semi-quantification was determined from the ratio of intensity between the band of the target virus and the band of the honey bee gene, *RpS5*, to determine the relative quantification units (RQUs) of viral RNA. To determine whether quantification at 35 amplification cycles was not affected by signal saturation of the band intensities, randomly selected samples with high, medium and low RQUs of DWV were also quantified in the same manner with 25 and 30 amplification cycles, and the RQU values were not significantly different for those samples whether 25, 30 and 35 amplification cycles were used (F_2,15_ = 0.30, P = 0.75).

### Statistical analysis

Data were visually examined for normality prior to statistical analyses using box plots. Paired comparisons of LMP and HMP colonies for daily mite fall counts, number of mites per 100 brood cells and number of mites per 100 adult bees were done using *t*-tests. Data on the number of mites per 100 brood or adults were square root-arcsine transformed, whereas the data for daily mite fall counts were subjected to log transformations because they were not normally distributed. *t* tests were also used to compare HMP and LMP bees of the different treatments for differences in relative amounts of viral RNA. Values presented are means ± standard error of the mean. All statistical analyses were performed with the R-Statistical Program [32].

## Results

### Mite levels in HMP and LMP colonies

Among 152 honey bee colonies, the ten LMP colonies chosen with a low difference between spring and summer in mean daily mite fall counts (approx. 4) was over 20 times less than the ten HMP colonies chosen with a high difference in mean daily mite fall counts for the same period (approx. 86). However, the mean daily mite fall counts between the LMP and HMP colonies in the spring (3.5 ±0.8 compared to 2.9 ±1.1, respectively) showed no significant differences (*P* >0.05). In contrast, significant differences were detected in summer (*P* <0.001) with mean daily mite fall counts of 7.8 ±1.4 for LMP colonies compared with 88.8 ±17.4 for HMP colonies.

Differences between LMP and HMP colonies in mean daily mite fall counts between spring and summer correlated well with the levels of infestation of brood (number of mites per 100 cells) and infestation of the adults (number of mites per 100 bees) in summer. There were significant differences between LMP and HMP colonies (*P* <0.01) for both the mean number of *V*. *destructor* per 100 cells (0.8 ±0.1 and 13.7 ±4.9 for LMP and HMP colonies, respectively) and the mean number of *V*. *destructor* per 100 worker bees (1.1 ±0.2 and 7.0 ±1.4% for LMP and HMP colonies, respectively).

### Viruses in mites and bees from HMP and LMP colonies

Mites used for infestation were collected from a colony with a mean daily mite fall count of 101 ±19.1 and no visible signs of virus symptoms among the bees. For three randomly selected samples of 100 *V*. *destructor* taken from this set of mites, DWV and IAPV were detected in each sample, but BQCV, ABPV, SBV and KBV were never detected.

**Fig 1:**
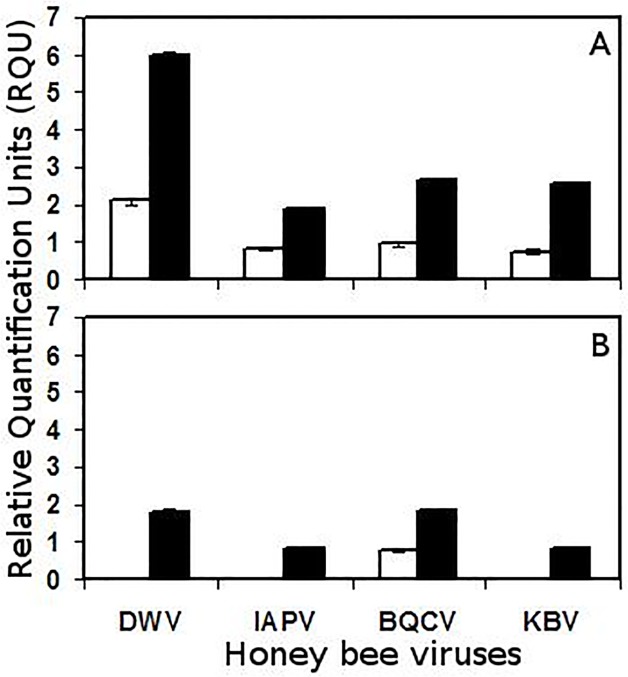
Relative RT-PCR product quantification of DWV, IAPV, BQCV and KBV by co-amplification with *A*. *mellifera* RpS5 in non-infested adult bees (white bars), or bees parasitized by *V*. *destructor* (black bars). (A) Relative viral levels in adult bees from a colony with high mite population growth (HMP). (B) Relative viral levels in adult bees from a colony with low mite population growth (LMP). Values presented are means ± se.

For non-infested adult bees, which were caged and treated like the infested adults but without the addition of the mite, DWV, IAPV, BQCV and KBV were detected from the selected HMP colony (Fig. 1). However, only BQCV was detected from non-infested adult bees from the LMP colony, although the relative BQCV level was similar to that in the HMP colony. For adult bees at 7 days infested with *V*. *destructor*, DWV, IAPV, BQCV and KBV were found in both the LMP and HMP colonies (Fig. 1). However, the relative amount of DWV, IAPV, BQCV and KBV were approx. 3 times, 2 times, 50% and 3 times higher, respectively, in mite infested adults from HMP compared to LMP colonies. Those differences were all significant (*P* <0.05). DWV occurred in significantly higher amounts than IAPV, BQCV and KBV in adults from the HMP colony, but that difference was much less for adults from the LMP colony, being only significantly higher than IAPV and KBV.

**Fig 2:**
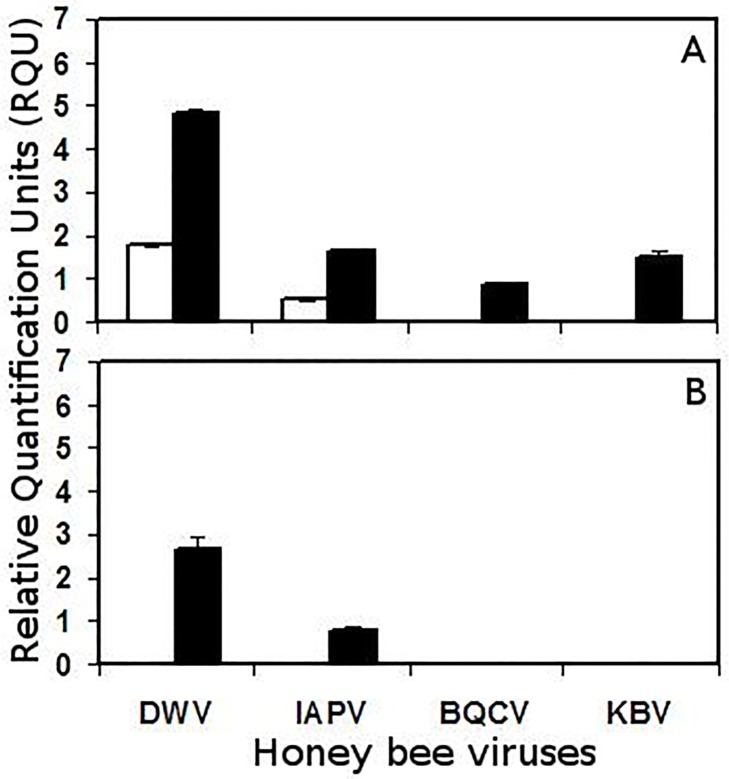
Relative RT-PCR product quantification of DWV, IAPV, BQCV and KBV by co-amplification with *A*. *mellifera* RpS5 in non-infested bee brood (white bars), or brood parasitized by *V*. *destructor* (black bars). (A) Relative viral levels in brood from a colony with high mite population growth (HMP). (B) Relative viral levels in brood from a colony with low mite population growth (LMP). Values presented are means ± se.

For non-infested brood, where the cells were opened and resealed as per mite infestation but without the addition of the mite, DWV and IAPV were detected in the HMP colony, whereas no viruses were detected in the LMP colony (Fig. 2). Seven days following infestation of brood with *V*. *destructor*, DWV, IAPV, BQCV and KBV were found in the HMP colony, but only DWV and IAPV were detected in the LMP colony (Fig. 2). However, the relative DWV and IAPV levels were approx. two times and 50% higher, respectively, in the brood from the mite infested HMP colony compared to the LMP colony, and both those differences were significant (*P* <0.05). DWV was much more common than the other viruses for brood from both the mite infested LMP and HMP colonies.

## Discussion

### Mite levels in HMP and LMP colonies

The difference in *V*. *destructor* population growth between HMP and LMP colonies was quite large (approx. 20 fold) from spring to summer. For these same colonies, Guzman-Novoa et al. [21] showed that bees from LMP colonies performed significantly more instances of intense grooming with a significantly higher number of mites being dislodged from their bodies in comparison to bees from HMP colonies. Also, Emsen et al. [20] showed that brood from these LMP colonies had a lower proportion of mites reproducing than brood in the HMP colonies. Although not examined by Emsen et al. [20], this was possibly due to *Varroa* sensitive hygienic behavior of adult bees because foundress mites that escape the removal process from cells by hygienic bees have a lower reproductive potential therefore reducing *V*. *destructor* population growth [33]. Thus, bees from these colonies appear to have differences related to both the ability of mites to reproduce and the degree of honey bee grooming behavior, traits that have been associated with honey bee resistance to *V*. *destructor*.

### Viruses in mites and bees from HMP and LMP colonies

Adults of the HMP colony appeared to be more susceptible to bee viruses than those of the LMP colony, even without exposure to mites. Four viruses were found in the control adults from the HMP colony versus only one virus in the control adults of the LMP colony. However, the relative amount of that virus (BQCV) did not differ between the adults of the HMP and LMP colonies. In addition, control brood of the HMP colony without exposure to mites appeared to be more susceptible to bee viruses than control brood of the LMP colony. Two viruses were detected in HMP brood compared to no viruses detected in LMP brood.

Following infestation with *V*. *destructor*, the same four viruses were detected in adults from both the HMP and LMP colonies. However, the relative amounts of those viruses were always significantly higher in adults from the HMP versus those of the LMP colony. There was also a difference in the occurrence of viruses in brood from HMP and LMP colonies infested with *V*. *destructor*. Four viruses were detected in brood from the HMP but only two from the LMP colonies. For the two viruses found in brood of both the HMP and LMP colonies, relative levels were always significantly higher in brood of the HMP colony. Thus, both adults and brood of the HMP colony with *V*. *destructor* appeared to be more susceptible to several bee viruses than those of the LMP colony.

The results with non-infested and mite-infested bees suggest that the lower virus levels in LMP brood and adults may have been due to epidemiological causes as bees from the LMP colony would have had less exposure to *V*. *destructor* and thus less parasitism by the mite with a correspondingly lessened chance for latent virus infections activated by parasitism by *V*. *destructor*. However, these results may also have an genotypic explanation; the LMP brood and adults may have had higher resistance to viral replication following transmission and/or activation of inapparent viruses.

Several honey bee viruses appear to be directly linked to the level of mite feeding. For example, there was a significant and rapid increase in DWV levels after brood or adult honey bees were exposed to *V*. *destructor* [34, 35], and DWV severity was positively correlated with the level of *V*. *destructor* parasitizing newly emerged honey bees using similar methods as in this study [36]. Because the amount of mite parasitism is reduced in the LMP colonies, then there would be less of a chance for the mite being able to transmit viruses in salival secretions and/or secrete immunosuppressive compounds that could activate latent infections in the honey bee. Small amounts of saliva of *V*. *destructor* secreted during parasitism have been proposed to activate DWV infections in honey bees through suppression of the bee's immune system [37]. Suppression of haemocyte-mediated wound healing in bees was proposed to occur when salivary secretions from *V*. *destructor* were shown to damage haemocytes [38]. Thus, if the much lower mite population growth found in the LMP colonies was due to reduced mite reproduction and possibly more intense grooming [20, 21], this could have resulted in reduced infections of *V*. *destructor*-transmitted viruses in the LMP colony for both infested and non-infested bees as a consequence of low mite parasitism. However, this possible explanation is less convincing for some viruses, such as IAPV, where its prevalence and titers have not been highly correlated with *V*. *destructor* levels [39].

By comparison, Locke et al. [19] took honey bee populations on the island of Gotland, Sweden, and identified colonies following natural selection that were mite resistant (MR) and compared them with a mite susceptible (MS) population of bees. Like the HMP and LMP colonies in this study, a comparison of adults from MR and MS colonies showed slower mite growth rates in the MR colonies, which was also related to some unknown adaptations that reduced reproduction of *V*. *destructor*. They showed that DWV levels increased similarly between MR and MS colonies from summer to autumn, except for slightly higher DWV levels in MR colonies in July. In contrast, BQCV and SBV levels decreased by several orders of magnitude in adults in MR versus MS colonies. Although KBV levels did not differ between adults of the MR and MS colonies, this was believed to be likely due to it being always at very low levels (i.e., near the detection limit). While the study by Locke et al. [19] and this study both found that BQCV levels differed significantly between the susceptible and resistant bees, this study also found that DWV and KBV levels were lower in bees from the colony with low rates of *V*. *destructor* population growth. One possible explanation for the different results is that there was natural selection for mite resistance in the Gotland populations for over a decade versus a one-time artificial selection for mite resistance in the Guelph populations.

Although we show a strong link between levels of *V*. *destructor* infestation and prevalence and levels of several viruses in bees from colonies selected for low and high mite population growth that also differ in the expression of traits related to mite resistance [20, 21], the experimental design and analyses of data of this study, cannot separate whether the effect is due to greater resistance to the viruses or to different levels of inapparent infections resulting from previous transmission related to high or low mite populations. Also, semi-quantification of virus is less accurate than real-time RT-PCR, although the differences observed in this study were large and reproducible. These matters can be addressed in future studies by using real-time RT-PCR and bidirectional selective breeding for mite population growth after several generations, to confirm underlying genetic effects linking mite resistance with virus resistance with and without mite infestation.
